# Innovative Joint for Cable Dome Structure Based on Topology Optimization and Additive Manufacturing

**DOI:** 10.3390/ma14185158

**Published:** 2021-09-08

**Authors:** Wenfeng Du, Hui Wang, Liming Zhu, Yannan Zhao, Yingqi Wang, Runqi Hao, Mijia Yang

**Affiliations:** 1 Institute of Steel and Spatial Structures, College of Civil Engineering and Architecture, Henan University, Kaifeng 475004, China; dwf0925@hotmail.com (W.D.); WangHui930815@163.com (H.W.); zyn931030@163.com (Y.Z.); WangYingqi666666@163.com (Y.W.); 2 Civil and Mechanical Engineering Laboratory, University of Rennes, 35000 Rennes, France; hmzhang999@126.com; 3 Department of Civil and Environmental Engineering, North Dakota State University, Fargo, ND 58102, USA; mijia.yang@ndsu.edu

**Keywords:** cable dome structure, support joint, topology optimization, smooth treatment, additive manufacturing

## Abstract

Aiming at the problems of a low material utilization rate and uneven stress distribution of cast-steel support joints in cable dome structures, topology optimization and additive manufacturing methods are used for optimization design and integrated manufacturing. First, the basic principle and calculation process of topology optimization are briefly introduced. Then, the initial model of the support joint is calculated and analyzed by using the universal software ANSYS Workbench 2020R2 and Altair OptiStruct, and the optimized joint is imported into Discovery Live to smooth the surface. The static behaviors of three types of joints (topology-optimized joints, joints after the smoothing treatment, and joints from practical engineering) are compared and analyzed. Finally, the joints are printed by using fused deposition modeling (FDM) technology and laser-based powder bed fusion (LBPBF) technology in additive manufacturing. The results show that the new support joint in the cable dome structure obtained by the topology optimization method has the advantages of a novel shape, a high material utilization rate, and a uniform stress distribution. Additive manufacturing technology can allow the manufacture of complex shape components with high precision and high speed. The combination of topology optimization and additive manufacturing effectively realizes the advanced design and integrated manufacturing of support joints for cable dome structures.

## 1. Introduction

The cable dome structure is a spatial structure system constructed by Geiger, an American engineer, based on the overall tension idea of Fuller [1,2]. It is a highly efficient full tension system. Since its first application in 1986 in the Seoul Olympic Fencing Gymnasium, it has been increasingly adopted in engineering fields, such as the Georgia Dome, Argentina La Plata football field, and Phoenix Mountain Sports Center in Chengdu, China [3]. The support joint is the most important part of the cable dome structure. The upper cable and lower cable usually meet here. If the support joint is damaged, the entire structure will be destroyed or even collapsed. Therefore, how to design and manufacture support joints with reasonable stress performance, safety, and reliability is particularly important [4].

In recent years, scholars and researchers have explored and researched cable dome structures. For example, Dong et al. [2,5] proposed a series of new cable dome structures, such as the Kiewitt type and Nest type, and their experimental research on the structural model provided a new structural form and theoretical basis for the design and application of cable domes. Zhang et al. [6] proposed a new structure called a steel-batten cable dome and discussed the construction technology of a semi-rigid steel batten cable dome. Chen et al. [7] studied the construction error influence and control technology of the first 100 m of a composite cable dome in China. However, most of these studies focus on new structural forms and the construction of prestressed cables, and there are few studies on the optimal design and manufacture of cable dome joints. To date, the design of cable dome support joints is still based on the experience of engineers; that is, the initial structural model is first established, and then the design scheme is determined through the cycle of analysis verification, optimization improvement, and remodeling. This process is time-consuming and laborious, the quality of joint design is uneven, and the problems of low material utilization and uneven stress distribution are prominent. In the production of the joint, the joint has been changed from welding manufacturing to integral casting, which effectively avoids the residual stress caused by the weld; however, the traditional casting process still faces the problems of low precision, long cycle, high cost, low efficiency, and serious environmental pollution [8].

To solve these problems, research can be performed from two aspects: optimal design and manufacturing method. On one hand, from the perspective of structural optimization [9,10], with the rapid development of mathematical optimization algorithms, topology optimization is becoming increasingly refined, which provides a new solution for structural optimization design. For example, Du et al. [11,12,13] studied the optimal shapes of two-branch joints, three-branch joints, and four-branch joints of treelike structures based on Optistruct software, and they attempted to use 3D printing technology to solve the problem that traditional casting methods could not easily form irregular shapes by topology optimization. Zhao et al. [14,15] performed topology optimization and 3D printing for a spatial structure joint based on the Optistruct software, discussed the influence of various optimization parameters on the results in detail, and combined the traditional process with 3D printing to realize the rapid production of joints. Zhang et al. [16] adopted a bionic substructure method to improve the effect of traditional topological optimization and applied it to the design of intersecting joints. Ye et al. [17] used the topology optimization method to study the rigid joint of a single-layer space grid structure and took a joint in an actual structure as an example to optimize the design. On the other hand, from the perspective of manufacturing methods [18,19], with the rapid development of additive manufacturing technology, scholars began to explore the feasibility of its application in building metal structures. Zhao [20] reviewed the mechanical properties, application fields, development, and current situation of 3D printing metal materials at home and abroad. Zhou et al. [21] used additive manufacturing technology to prepare 316L stainless steel standard parts and performed tensile property tests. He et al. [22] studied the 3D modeling and printing of tree-like structure joints and verified the mechanical properties of 3D-printed joints through finite element simulation and experimental study. Combining the two aspects, topology optimization is an effective way to realize the optimal design of joints, and additive manufacturing can quickly manufacture high-precision complex joints. Combining the two aspects, topology optimization is an effective method to optimally design joints, and additive manufacturing can quickly manufacture high-precision complex joints such as cable dome support joints.

In this paper, based on the engineering background of an actual cable dome structure, optimization design and integrated manufacturing research are performed by using topology optimization and additive manufacturing methods. First, an initial model of cast-steel joints is established. Under the boundary constraints and symmetrical constraints, topology optimization is performed with the minimum flexibility (maximum stiffness) as the objective, and the optimized geometry of the joint is obtained. Then, Discovery Live is employed to smooth the topology-optimized joint, and the static behaviors of the smoothing joint, topology-optimized, joint and actual joint based on experience design in engineering practice are compared and analyzed. Finally, additive manufacturing technology is used to replace the traditional casting process to manufacture these joints.

## 2. Topology Optimization Method and Mathematical Model

### 2.1. Topology Optimization Method

**Figure 1:**
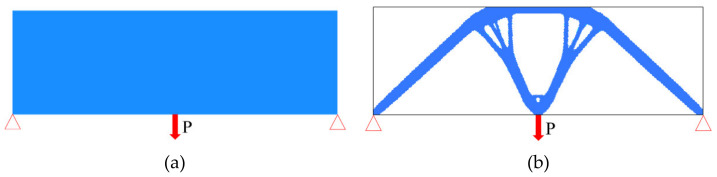
Illustration of topology optimization: (**a**) design area and boundary conditions; (**b**) result of the topology optimization.

Topology optimization is a mathematical method that optimizes the material distribution in the given design area according to the boundary conditions and performance indices. It can automatically find the optimal distribution of materials under given conditions, as shown in Figure 1, and obtain optimal mechanical properties with the least materials.

The cast-steel joints of the cable dome support studied in this paper belong to the continuum of structure topology optimization, and the most common method is solid isotropic material with penalization model (SIMP). The SIMP discretizes the design area into finite elements and sets the relative density *ρ* of each element to be 0–1. *ρ* = 0 indicates that the element is in the state of no material filling, and *ρ* = 1 indicates that the element is in the state of full material. In most cases, the relative density of the element is in between. To make the material more clearly show the presence or absence of two states, the stiffness and Young’s modulus of the element are expressed by the relative density as an exponential relationship.

*k = ρ^P^k_0_* (1)

*E = ρ^P^E_0_* (2)

where *K*_0_ is the element stiffness matrix when the element is filled with material; *E*_0_ is Young’s modulus of elasticity when the element is filled with material; *P* is the penalty factor, which is usually taken as 3.0. The penalty factor is introduced to make the relative density of cells gather to 0 or 1 as much as possible.

### 2.2. Mathematical Model

The three elements of optimization design are design variables, constraints, and objective functions. Design variables are shifting parameters. The boundary condition is the constraint on the structure. The objective function is to achieve the optimal design performance [23]. The mathematical expression is as follows:

Design variable:

*f_i_*(*x*), (*i* = 1, 2, 3, …, *M*) (3)

Constraint condition:

*G_j_*(*x*), (*j* = 1, 2, 3, …, *M*) (4)

Objective function:

*H_k_*(*x*), (*k* = 1, 2, 3, …, *M*). (5)

Since the cable dome support joint provides rigid support for the entire cable dome structure, topology optimization selects the maximum stiffness (minimum flexibility) as the goal. To achieve maximum stiffness with minimum material, the volume fraction of the joint is taken as the constraint, and the design variable of the model is the element density [24,25]. Therefore, the mathematical model of topology optimization can be described as follows:

*min C*(*x*) = 1/2*U^T^KU* *s.t. F* = *KU*
*V*(*x_i_*) ≤ *V*^+^
0 < *x_min_* ≤ *x_i_* < *x_max_* ≤ 1 (6)

where *N* is the total number of finite elements of the entire joint; *C* is a function of *X* and represents the flexibility of the joint; *U* is the structural displacement; *K* is the overall stiffness of the joint; *V*^+^ is the volume fraction of the joint.

### 2.3. Topology Optimization Process

**Figure 2:**
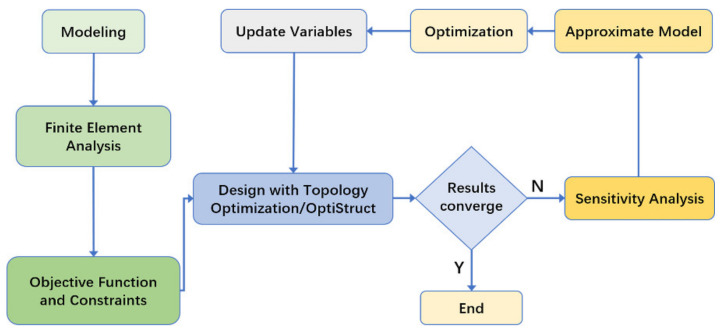
Topology optimization process.

In this paper, the topology optimization module of ANSYS Workbench 2020R2 (ANSYS, Inc., Pittsburgh, PA, USA) is employed to optimize the joint, and the results are compared with those of OptiStruct in Altair HyperWorks (Altair Engineering Inc., Troy, MI, USA). ANSYS has a similar topology optimization process to Altair, as shown in Figure 2. Under the given constraints, the material distribution is changed by the optimization algorithm. The optimization calculation is performed under the given design objective. When the difference of three successive iterations of the objective function is lower than the given tolerance, the topology optimization results converge.

## 3. Topology Optimization of Cable Dome Support Joint

### 3.1. Initial Structural Model

**Figure 3:**
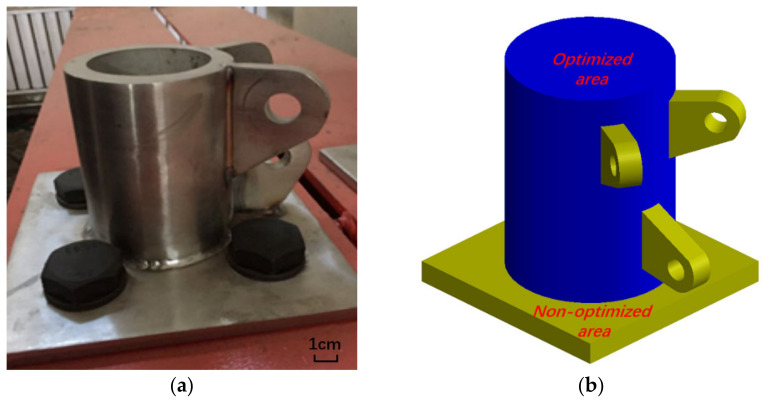
Initial support joint: (**a**) scale test model of the practical engineering joint, (**b**) initial finite element model of the joint.

Referring to the cast-steel support joint of the cable dome structure in actual engineering, as shown in Figure 3a, the initial model of the support joint of the cable dome structure is established by using SolidWorks 2019 (Dassault Systemes Inc., Waltham, MA, USA) software, as shown in Figure 3b.

**Figure 4:**
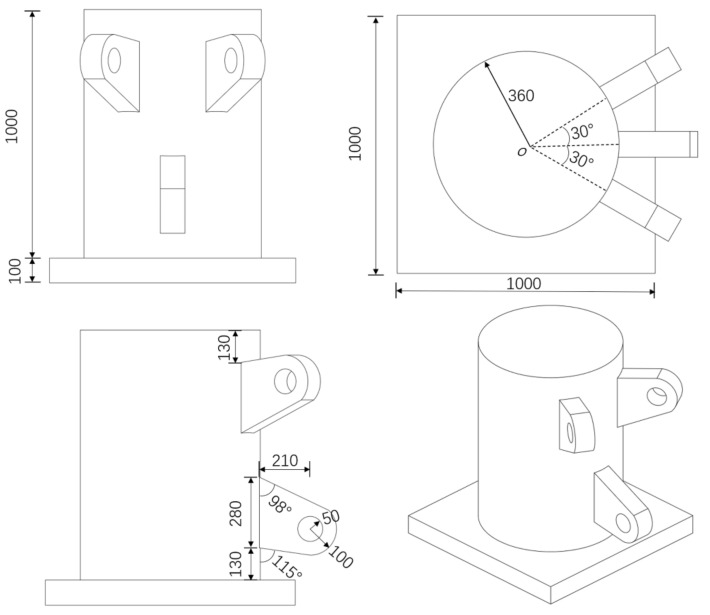
Geometry size of support (unit: mm).

The initial finite model is divided into an optimized area (blue region) and non-optimized areas (yellow regions). The optimized area is a cylindrical solid, the topology optimization analysis is performed in this part, and the material distribution density is optimized to obtain the geometric optimal shape of the joint. The non-optimized areas include the bottom plate and three ear plates, which are determined as geometry-preserving entities and do not participate in the optimization iterative calculation. Figure 4 shows the geometry size of the support joint.

### 3.2. Finite Element Analysis of the Initial Joint

**Figure 5:**
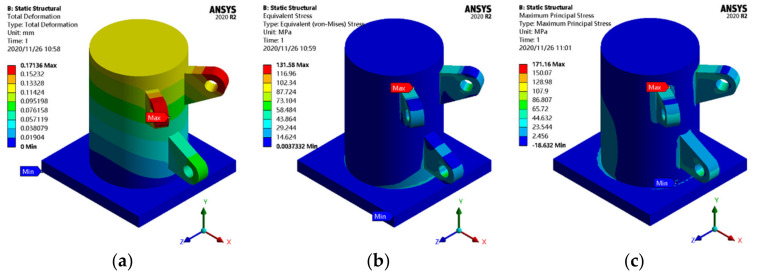
Static analysis results of the initial joint: (**a**) displacement cloud map; (**b**) equivalent stress cloud map; (**c**) principal stress cloud map.

The joint model is output using x_t format and imported into the ANSYS Workbench 2020r2 software. Then, the material properties of the model are provided. In this paper, the joint of the cable dome support is a cast-steel joint, the elastic modulus is 206,000 MPa, the Poisson’s ratio is 0.3, the density is 7.85g/cm^3^, and the mass of the joint is approximately 3.2 t. In the mesh module, if the mesh size is large, the results of the finite element simulation are distorted. On the contrary, it will consume a very long time in finite element analysis and topology optimization. We tried meshing with different sizes, and it is appropriate for us to control the mesh size at 0.02 m, which also prevents the adverse effect caused by the large difference of mesh size in the optimization area. In total, 835,858 nodes and 599,220 elements are obtained. Finally, the boundary conditions are assigned, where the base plate of the support is set as a fixed constraint, and a concentrated force of 500 kN is applied on the circular hole surface of each ear plate, whose direction is along the tangent direction of the upper edge of the ear plate. After the preprocessing of finite element analysis, the static behavior is analyzed, and the results are shown in Figure 5.

The static analysis results show that the maximum displacement, maximum equivalent stress, and maximum principal stress of the initial joint model are 0.17 mm, 131.58 MPa, and 171.16 MPa, respectively. The maximum displacement appears at the outer edge of the ear plate, and the maximum stress and maximum principal stress appear at the junction of the ear plate and optimization area. Notably, the static nephogram shows that the stress distribution of the joint is uneven, the overall utilization rate of the material is very low, and most of the materials are in a state of low stress. Therefore, the topology optimization method must be used to optimize the design to obtain a more reasonable geometric configuration.

### 3.3. Topology Optimization by ANSYS

**Figure 6:**
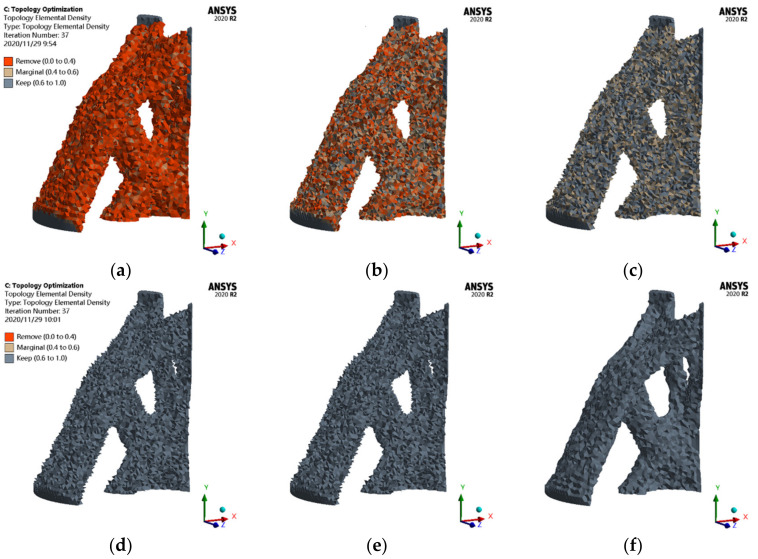
Isosurface map of ANSYS: (**a**) *ρ* ≥ 0.1; (**b**) *ρ* ≥ 0.3; (**c**) *ρ* ≥ 0.5; (**d**) *ρ* ≥ 0.7; (**e**) *ρ* ≥ 0.9; (**f**) *ρ* = 1.0.

Topology optimization by ANSYS Workbench is based on the finite element static analysis. After the static analysis, some parameters are set. The maximum number of iteration steps is set to 500, and the minimum normalized density is 0.001. To avoid checkerboard phenomena, the penalty factor is set to 3. Then, the optimization area and non-optimization area are defined, and the maximum stiffness (minimum flexibility) is defined as the objective function. With a volume fraction of 13% as the constraint, convergence is defined when the convergence accuracy reaches 0.1%; i.e., when the objective difference of any three consecutive iterations is less than 0.1%, the topology optimization solution converges. After 37 iterations in ANSYS, the convergence of topology optimization calculation of the cable dome support joint is completed. The isosurface map of the ANSYS topology optimization is shown in Figure 6.

When the element density is *ρ* ≥ 0.1, the output result is as follows: the shape of the entire cast-steel joint with an element density below 0.1 is removed by the topology optimization program, and the retained shape reflects the general shape of the topology optimization result. When the element density *ρ* ≥ 0.3, the optimization algorithm continues to remove the element whose density is 0.1–0.3, and the topology optimization shape is clearer. Similarly, when the element density further increases, less material remains after optimization, and the degree of optimization increases. Comparing the isosurface map corresponding to different element densities, we observe that the structural shape is relatively close after the element density *ρ* ≥ 0.3 due to the high utilization rate of this part of materials; i.e., the element density corresponding to the element on the stress path is larger, and less deletion is required after each iteration. 

ANSYS divides the element density into three levels, which are the parts that must be removed when the element density is 0.4. The edge area (0.4–0.6) is adjusted by the designer to decide which element density area should be retained and which element density area should be deleted. The area with an element density of 0.6–1.0 is the reserved area.

### 3.4. Topology Optimization by OptiStruct

The commercial software of continuum topology optimization based on the variable density method has been developed in many large-scale general finite element software programs. To compare and verify the topology optimization results of ANSYS Workbench, OptiStruct in Altair HyperWorks is employed for the topology optimization analysis.

**Figure 7:**
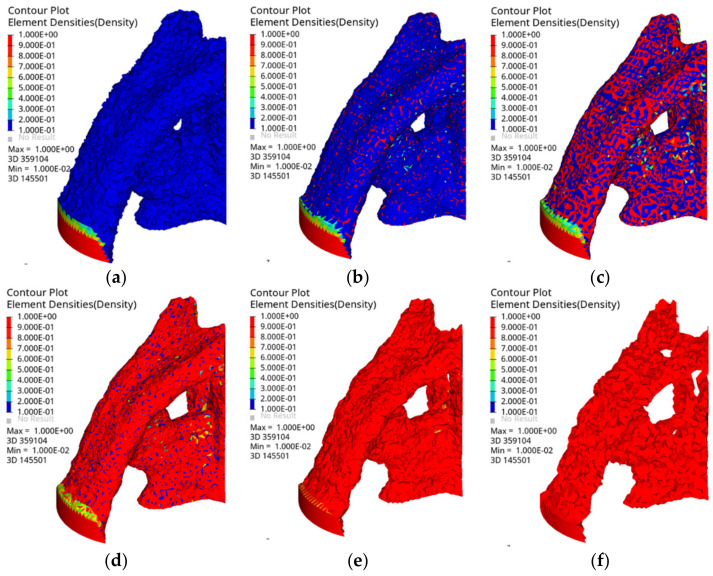
Isosurface map of OptiStruct: (**a**) *ρ* ≥ 0.1; (**b**) *ρ* ≥ 0.3; (**c**) *ρ* ≥ 0.5; (**d**) *ρ* ≥ 0.7; (**e**) *ρ* ≥ 0.9; (**f**) *ρ* = 1.0.

The parameter setting of OptiStruct is identical to that of ANSYS. After 28 iterations, the topology optimization result converges. Figure 7 shows the isosurface map of the OptiStruct results. By default, OptiStruct divides the element density from (0, 1) into ten intervals, which are represented by different colors. To more clearly compare the differences between two software programs, two types of topology calculation results are compared and discussed.

**Figure 8:**
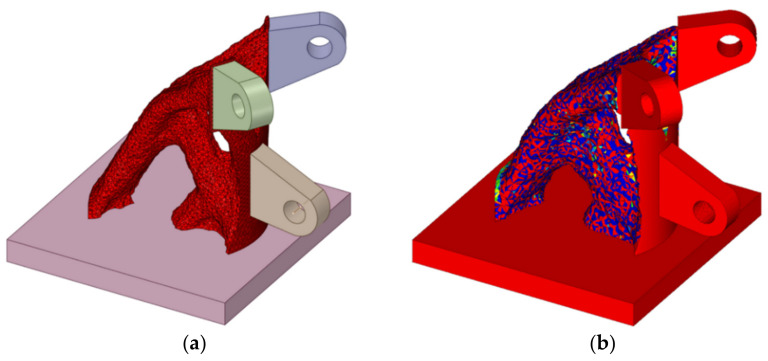
Topology-optimized joint: (**a**) topology-optimized joint by ANSYS; (**b**) topology-optimized joint by Optistruct.

Comparing the topology optimization results of ANSYS and OptiStruct, we observe that the two software programs have similar analysis results, and the topology optimization forms are essentially identical. For both ANSYS and OptiStruct, the areas with element density of 0–0.4 are in the state of low material filling density and must be deleted, while those with element density of 0.6–1 have higher material filling density and must be retained. In addition, ANSYS shows the areas of element density that must be reserved or removed. Optistruct shows different element densities and different colors, and it can more intuitively and clearly display the filling state of each part of the material. In this paper, we compare the results of element densities *ρ* ≥ 0.4, *ρ* ≥ 0.5, and *ρ* ≥ 0.6 in ANSYS and Optistruct and choose the model of element density *ρ* ≥ 0.5 as the topology-optimized joint. Figure 8 shows the joint model after the topology optimization.

After the topology has been optimized, the mass of the joint is approximately 1.17 t. Compared with the initial joint, the mass is reduced by approximately 52.4%, which greatly reduces the amount of materials. According to the optimized shape characteristics, the materials are concentrated near the force transmission path, and the topology-optimized joint is novel, reasonable, and creative.

### 3.5. Smooth Treatment

In both ANSYS and OptiStruct topology optimization results, there is a common defect: the structure surface after topology optimization is uneven, which does not satisfy the visual needs of people for building aesthetics. This is an inherent defect based on finite element calculations. Therefore, how to quickly smooth the topology optimization results is the key problem to solve.

**Figure 9:**
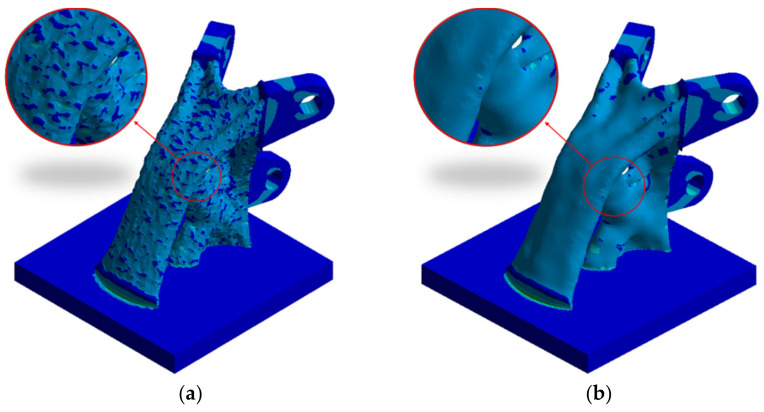
Comparison of the joint surface before and after smoothing: (**a**) before smoothing; (**b**) after smoothing.

In this paper, the Discovery Live software is used to quickly smooth the topology optimization results. First, the topology-optimized joint is imported into the Discovery Live software. Then, we must select the module of topology optimization and define the simulation subject of topology optimization, which is the part that must be smoothed. According to the boundary conditions of static analysis of the original model, the boundary conditions of the model are set. The volume reduction of topology optimization is set to 0, and the structure is calculated. When the calculation is completed, we select Smooth and save it. Figure 9 shows the comparison of the joint surface before and after smoothing.

## 4. Comparison Analysis and Discussion

**Figure 10:**
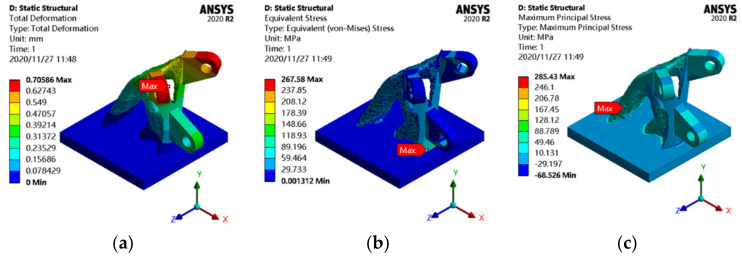
Static analysis results of the topology-optimized joint: (**a**) displacement cloud map; (**b**) equivalent stress cloud map; (**c**) principal stress cloud map.

To investigate the difference in static behavior between topology-optimized joints and other joints, a finite element analysis of topology-optimized joints was performed. The joint is applied with identical constraints and external loads as the initial joint. The static analysis results of the topology-optimized joint are shown in Figure 10.

According to Figure 10, the maximum displacement of the topology-optimized joint is 0.71 mm, which appears at the outer edge of the ear plate. The maximum equivalent stress is 267.58 MPa, which is different from that of the initial joint. The maximum equivalent stress appears at the junction of the edge near the ear plate and the x-axis direction of the bottom plate. The maximum principal stress is 285.43 MPa, which occurs at the junction of the x-axis direction of the bottom plate and the edge of the optimization area far away from the ear plate.

**Figure 11:**
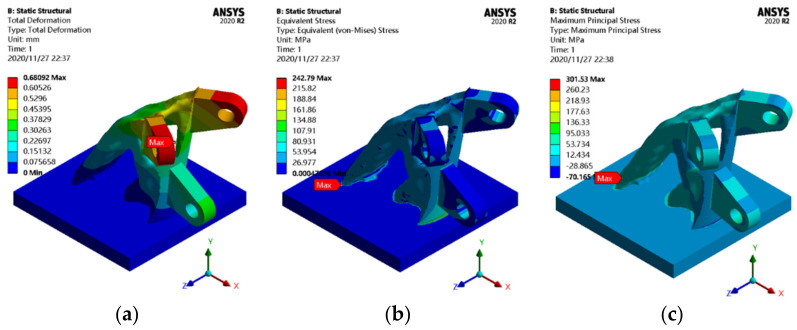
Static analysis results of the smoothing joint: (**a**) displacement cloud map; (**b**) equivalent stress cloud map; (**c**) principal stress cloud map.

To investigate the effect of the smooth treatment on the mechanical properties of the joint, Figure 11 shows the static analysis results of the joint after smoothing.

The mass of the smoothing joint is approximately 1.17 t, which is equal to that before smoothing, and the shape of the joint before and after smoothing has not changed, but the rough surface of the joint completely disappears. Compared with the static behavior analysis results of the topology-optimized joint, the maximum displacement of the smoothed structure is reduced by approximately 0.03 mm, which still appears at the outer edge of the ear plate. The maximum equivalent stress is 242.79 MPa, which decreases by 24.79 MPa compared to that before smoothing, and the position is shifted to the junction of the side tips and bottom plate of the optimization area. The results of the static behavior analysis show that the stress of the joint is more uniform due to the reduction of the sharp surface after the joint has been smoothed.

To comprehensively discuss the practical effect of topology optimization, the static analysis results of topology-optimized joints and smoothing joints are compared with actual joints based on experience design in engineering. The actual joint is established with to a certain project (Figure 3a), which is a hollow cylinder model with a wall thickness of 50 mm, and other geometric characteristics are identical to those of the initial joint.

**Figure 12:**
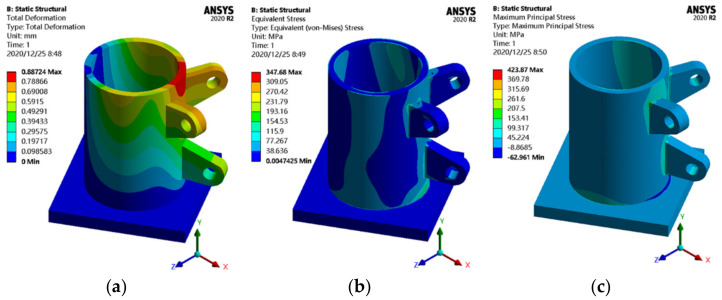
Static analysis results of the actual joint: (**a**) displacement cloud map; (**b**) equivalent stress cloud map; (**c**) principal stress cloud map.

Figure 12 shows the results of the finite element static behavior analysis of the actual joint. The mass of the actual joint is approximately 1.75 t, and the maximum displacement is 0.89 mm, which appears at the edge of the circular steel tube in the middle of the upper ear plate. The maximum equivalent stress is 347.68 MPa, and the maximum principal stress is 423.87 MPa, which are located at the upper part of the junction between the upper ear plate and the steel pipe.

**Figure 13:**
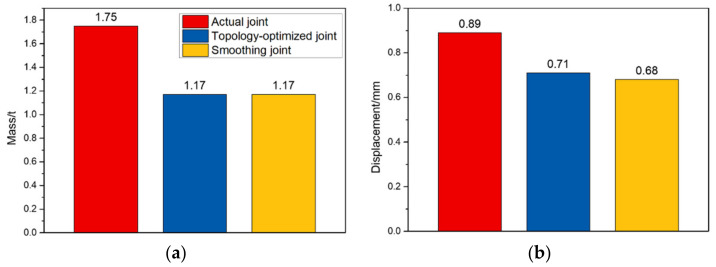

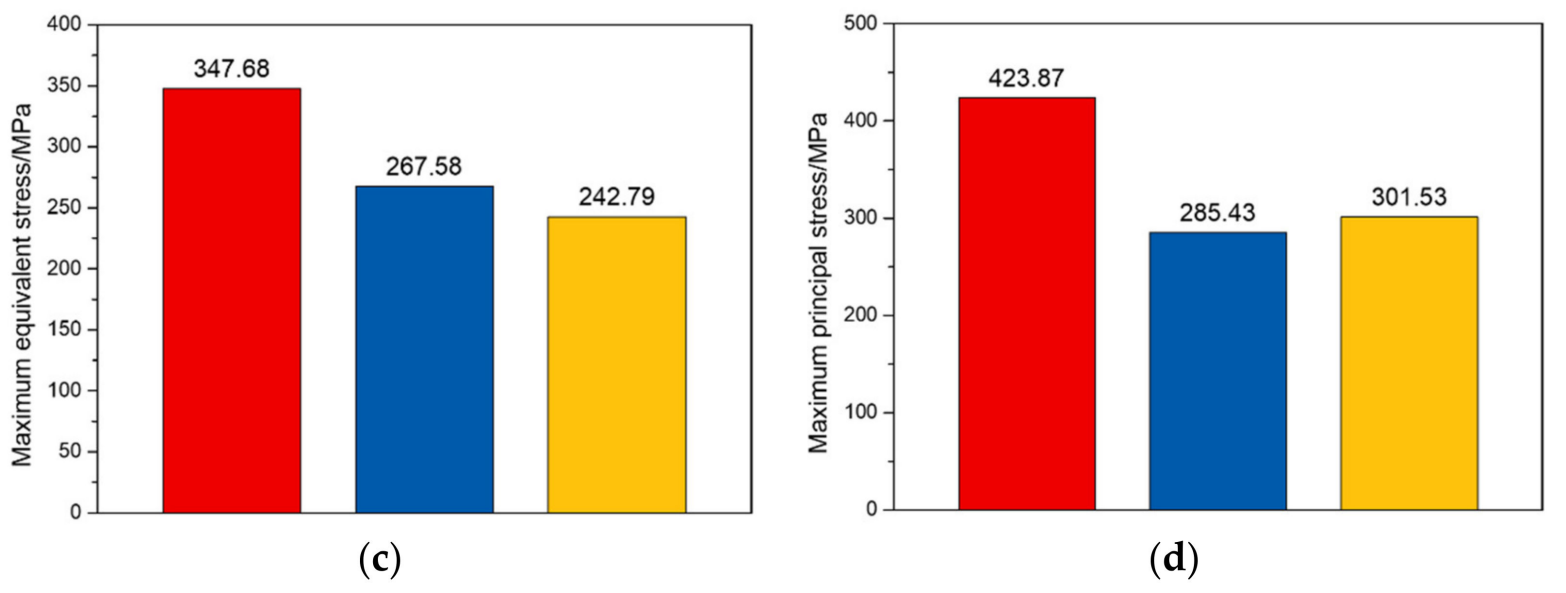
Histogram of the joint behavior: (**a**) mass; (**b**) displacement; (**c**) maximum equivalent stress; (**d**) maximum principal stress.

In summary, the static behaviors of smoothing joints, topology-optimized joints, and actual joints are compared in detail, and the actual joint is taken as the reference. The comparison results are shown in Figure 13 and Table 1.

Figure 13 and Table 1 show that the effect of topology optimization is very significant. Compared with the actual joint, the mass of the topology-optimized joint is reduced by 33.14%, while its static behavior is improved. The maximum displacement, maximum equivalent stress, and maximum principal stress are reduced by 20.22%, 23.04%, and 32.66%, respectively. Moreover, the smoothing joint has better static behaviors than the topology optimization. Compared with the actual joint, its mass, maximum displacement, maximum equivalent stress, and maximum principal stress are reduced by 33.14%, 23.60%, 30.17%, and 28.86%, respectively. In general, the topology optimization technology achieves the goal of optimization design. To ensure the static behavior, the topology optimization technology maximizes the utilization efficiency of materials and reduces the material consumption. Combined with the smoothing method, it solves the problem of surface roughness of traditional topology optimization results and makes the surface stress of topology-optimized joints more uniform. The topology optimization technology provides an effective method to optimally design cable dome support joints.

## 5. Additive Manufacturing

Additive manufacturing (AM), which is known as 3D printing, is a revolutionary method of industrial production. It is based on the CAD model or digital model and uses powdered adhesive materials to construct objects by stacking layer by layer [26,27,28]. At present, the main additive manufacturing methods are laser-based powder bed fusion (LBPBF), fused deposition modeling (FDM), arc additive manufacturing (WAAM), and stereolithography appearance (SLA) [29,30]. In recent years, experts and scholars have attempted to apply additive manufacturing technology to manufacture structural joints. In the additive manufacturing process, the CAD model or digital model must be converted into a virtual code to manufacture various joints. Additive manufacturing technology improves the manufacturing speed and precision and solves the problem that joints with complex shapes are difficult to produce by traditional processes.

### 5.1. Preparation for Printing

At present, the performance of a product made at different scales may vary due to, among other things, the distribution of microstructure defects resulting from the additive technology itself. It is highly consuming to achieve the full-scale additive manufacturing of large models. Consequently, all joints in this paper were reduced by 10 times for 3D printing. FDM and LBPBF 3D printers from the structural laboratory of Henan University were employed to fabricate the joints. The materials were 316L stainless steel (SS) and renewable biodegradable material polylactic acid (PLA).

**Figure 14:**
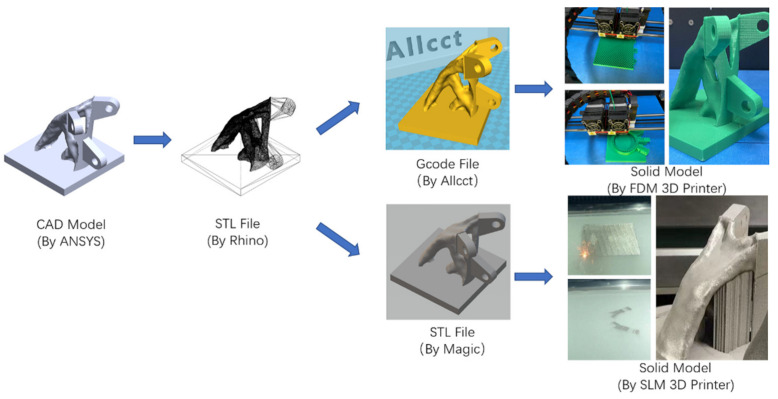
Additive manufacturing of the smoothing joint (images by Hui Wang).

The smoothing joint, topology-optimized joint, and actual joint are scaled by 0.1. Then, the CAD model file is transformed into an STL file in ASCII code. The model is sliced by a special software to form a G code file, which is recognizable by a 3D printer, and imported into a 3D printer for the additive manufacturing of all joints. The additive manufacturing process for smoothing joints is shown in Figure 14.

### 5.2. Printed Products and Discussion

**Figure 15:**
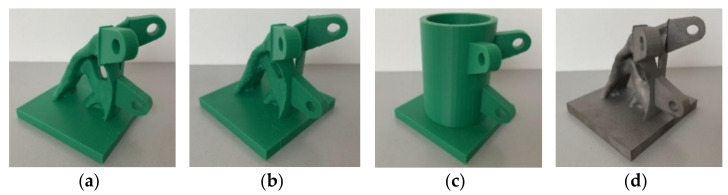
Solid models printed by a 3D printer: (**a**) smoothing joint; (**b**) topology-optimized joint; (**c**) actual joint; (**d**) LBPBF smoothing joint (Images by Hui Wang).

Figure 15 shows the smoothing joint, topology-optimized joint, and actual joint successfully manufactured by FDM printer. Figure 15d shows the SS joint made by the LBPBF method.

As shown in Figure 15, the additive manufacturing technology has high joint precision and overall effect. In addition, because of the principle of lamination printing and superposition, the additive manufacturing technology does not require separate molds, so it can easily and rapidly manufacture complex shape structures. From the production time viewpoint, the traditional casting method requires nearly half a month to complete a series of complicated and time-consuming processes, whereas additive manufacturing metal joints only take 20 h.

**Figure 16:**
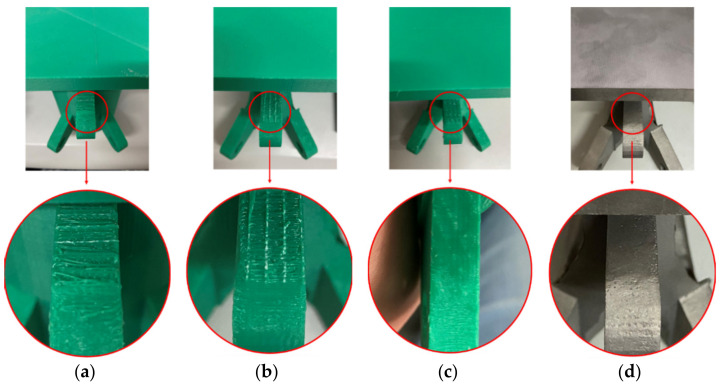
Influence of different support densities on the supporting surface: (**a**) 5% support; (**b**) 10% support; (**c**) 15% support; (**d**) LBPBF 15% support (Images by Hui Wang).

After dozens of printing experiments, we have found that a reasonable parameter setting is the key to obtaining high-precision joints. To improve the printing quality, it is necessary to optimize the process parameters, such as the layer thickness, filling density, temperature, power, and speed. The layer thickness is the printing thickness of each layer along the height direction. If the thickness is less than 0.1 mm, the product quality is very high, but when the layer height is greater than 0.2 mm, the product quality will be low. The filling density is the filling density of internal materials in the printing process. For the FDM printer, a 20% material filling rate is appropriate. For the LBPBF printer, to more truly restore the actual joint, the material filling rate is set to 100%. The maximum printing speeds of the FDM and LBPBF printers in this experiment were 150 mm/s and 6 m/s, respectively. For FDM printers, if the printing speed is too high, the model may not print because the extruded materials cannot keep up with the printing speed. If the setting speed is low, the material of the nozzle may be blocked. After many tests, the suitable printing speed of the FDM printer was set to 40 mm/s, and the suitable printing speed of the LBPBF printer was set to 700 mm/s. Since the cantilever part falls under the action of gravity in the printing process, it needs support in the branch. The support mode and support density significantly affect the printing quality, as shown in Figure 16. When there is no support, printing failure easily occurs because the material is suspended. Figure 16a,b show that when the support density is 5% and 10%, the printing process can be completed, but the surface is rough. When the joint is printed with a 15% support density, the surface of the suspended part of the model is relatively smooth, as shown in Figure 16c. The same problem exists when printing metal joints, as shown in Figure 16d. The setting of the support mode and support density must be determined according to the specific shape of the printing structure, mainly considering the balance of the extended part of the main body under the influence of gravity. Table 2 summarizes the optimal setting of printer parameters in this printing process.

## 6. Conclusions 

To solve the problems of the low material utilization rate and uneven stress distribution of cast-steel support joints in cable dome structures, the topology optimization method was used to study the support joints of engineering cable dome structures. Then, the static behaviors of the smoothing joint, topology-optimized joint, and actual joint are compared. Finally, the joints are 3D-printed using the additive manufacturing technology. The main conclusions are as follows:

(1) Compared with the actual joint, the mass of the topology-optimized joint is reduced by 33.14%, and the static behaviors are improved by approximately 20%. It is feasible to explore a reasonable structural form of cast-steel support joints of cable dome structures through topology optimization, which can satisfy the mechanical properties and significantly improve the utilization rate of materials.

(2) The smooth treatment is effective to solve the problem of rough surfaces of topology optimization results. This method retains the shape of topology-optimized joints and improves the static behaviors.

(3) additive manufacturing can compensate for the defects of traditional casting processes, such as low precision, slow speed, and difficulty in realizing complex shapes, which satisfies the requirements of current intelligent manufacturing.

(4) Topology optimization, smooth treatment, and additive manufacturing can be combined to effectively realize the optimal design and integrated manufacturing of cast-steel support joints of cable dome structures.

## Figures and Tables

**Table 1:** Comparison of three types of joints.

Joint	Actual Joint	Topology-Optimized Joint		Smoothing Joint	
	Raw Data	Raw Data	Comparison	Raw Data	Comparison
Mass/t	1.75	1.17	−33.14%	1.17	−33.14%
Displacement/mm	0.89	0.71	−20.22%	0.68	−23.60%
Maximum equivalent stress/MPa	347.68	267.58	−23.04%	242.79	−30.17%
Maximum principal stress/MPa	423.87	285.43	−32.66%	301.53	−28.86%

**Table 2:** Summary of the additive manufacturing parameters.

Printing Type	Material	Layer Thickness (mm)	Edge Thickness (mm)	Filling Density	Printing Temperature/Power	Printing Speed (mm/s)	Support Mode
FDM	PLA	0.2	0.8	20%	210 °C	50	All support
LBPBF	Austenitic stainless steel	0.05	-	100%	150 W	700	Local support

## Data Availability

Some or all of the data, models, or code that support the findings of this study are available from the corresponding author upon reasonable request.
